# Recommendations of the Advisory Committee on Immunization Practices for Use of a Hepatitis B Vaccine with a Novel Adjuvant

**DOI:** 10.15585/mmwr.mm6715a5

**Published:** 2018-04-20

**Authors:** Sarah Schillie, Aaron Harris, Ruth Link-Gelles, José Romero, John Ward, Noele Nelson

**Affiliations:** ^1^Division of Viral Hepatitis, National Center for HIV/AIDS, Viral Hepatitis, STD, and TB Prevention, CDC; ^2^Pediatric Infectious Diseases Section, University of Arkansas for Medical Sciences and Arkansas Children’s Hospital, Arkansas Children’s Hospital Research Institute.

Hepatitis B (HepB) vaccination is the primary means of preventing infections and complications caused by hepatitis B virus (HBV). On February 21, 2018, the Advisory Committee on Immunization Practices (ACIP) recommended Heplisav-B (HepB-CpG), a yeast-derived vaccine prepared with a novel adjuvant, administered as a 2-dose series (0, 1 month) for use in persons aged ≥18 years. The ACIP Hepatitis Vaccines Work Group conducted a systematic review of the evidence, including data from four randomized controlled trials assessing prevention of HBV infection and six randomized controlled trials assessing adverse events in adults. Seroprotective antibody to hepatitis B surface antigen (anti-HBs) levels were achieved in 90.0%–100.0% of subjects receiving HepB-CpG (Dynavax Technologies Corporation), compared with 70.5%–90.2% of subjects receiving Engerix-B (GlaxoSmithKline Biologicals). The benefits of protection with 2 doses administered over 1 month make HepB-CpG an important option for prevention of HBV.

## Introduction

Vaccination is the primary means for preventing hepatitis B virus (HBV) infection and its complications. Existing hepatitis B (HepB) vaccines use an aluminum adjuvant. On November 9, 2017, Heplisav-B (HepB-CpG), a single-antigen HepB vaccine with a novel immunostimulatory sequence adjuvant, was approved by the Food and Drug Administration for the prevention of HBV in persons aged ≥18 years. The vaccine is administered as 2 doses, 1 month apart (*1*). On February 21, 2018, the Advisory Committee on Immunization Practices (ACIP)* recommended HepB-CpG for use in persons aged ≥18 years.

HepB-CpG contains yeast-derived recombinant HepB surface antigen (HBsAg) and is prepared by combining purified HBsAg with small synthetic immunostimulatory cytidine-phosphate-guanosine oligodeoxynucleotide (CpG-ODN) motifs (1018 adjuvant). The 1018 adjuvant binds to Toll-like receptor 9 to stimulate a directed immune response to HBsAg (*1*).

HepB-CpG is available in single-dose 0.5 mL vials. Each dose contains 20 *μ*g of HBsAg and 3,000 *μ*g of 1018 adjuvant. HepB-CpG is formulated without preservatives and is administered as an intramuscular injection in the deltoid region of the upper arm (*1*).

HepB-CpG is the fifth inactivated HepB vaccine currently recommended for use in the United States. This report contains ACIP guidance specific to HepB-CpG and augments the 2018 ACIP recommendations for the prevention of HBV infection (*2*). This report does not include new guidance for populations recommended to receive HepB vaccination or immunization management issues other than those that pertain specifically to HepB-CpG. The intended audience for this report includes clinical and public health personnel who provide HepB vaccination services to adults. These recommendations are meant to serve as a source of guidance for health care providers; health care providers should always consider the individual clinical circumstances of each patient.

## Methods

From February 2016 to January 2018, the ACIP Hepatitis Vaccines Work Group^†^ participated in three teleconference meetings to review the quality of evidence for immunogenicity and safety of HepB-CpG and implementation issues. The Grading of Recommendations Assessment, Development and Evaluation (GRADE) approach for evaluating evidence was adopted by ACIP in 2010 (https://www.cdc.gov/vaccines/acip/recs/grade/). The Work Group identified critical and important outcomes for inclusion in the GRADE tables, conducted a systematic review of the evidence, and subsequently reviewed and discussed findings and evidence quality (*3*). Key outcomes were designated as critical (hepatitis B infection, severe adverse events, and cardiovascular safety) or important (mild adverse events). Factors considered in determining the recommendation included benefits and harms and evidence type. Values and preferences and economic factors were not systematically considered.

The scientific literature was searched through a systematic review of Medline (Ovid), CAB Abstracts, Embase, Global Health (Ovid), Scopus, and Cochrane databases. Search terms included “Heplisav,” “HBV-ISS,” “HBsAg-1018,” “1018 immunostimulatory sequence,” and “hepatitis B surface antigen-1018 ISS.” To qualify as a candidate for inclusion in the review, a study had to present immunogenicity or disease endpoints or safety data on HepB-CpG. Studies were excluded if they were basic science, a secondary data analysis, immunogenicity outcomes for a nonlicensed formulation or use of HepB-CpG, a general review or opinion perspective, conducted on nonhuman primates, or if data could not be abstracted. Supporting evidence for the Work Group’s findings is available online (https://www.cdc.gov/vaccines/acip/recs/grade/hepb.html).

A summary of Work Group discussions was presented to ACIP on October 25, 2017, and February 21, 2018. At the February 2018 meeting, a proposed recommendation was presented to the committee, and, after a public comment period, was approved by the voting ACIP members as follows: HepB-CpG is recommended as an option for HepB vaccination for persons aged ≥18 years (14 voted in favor, with none opposed, none abstained, and none recused). This report summarizes the data considered, the quality of evidence, and the rationale for the recommendation.

## Summary of Key Findings

The body of evidence consisted of four randomized controlled trials assessing prevention of HBV infection and six randomized controlled trials assessing adverse events (mild adverse events, serious adverse events, and cardiovascular adverse events) in adult subjects. Outcomes compared HepB-CpG with Engerix-B. Data from these studies informed HepB-CpG licensure. Studies assessing prevention of HBV infection used antibody to hepatitis B surface antigen (anti-HBs) ≥10 mIU/mL as a serologic correlate of protection. Protection among 7,056 subjects receiving 2 doses of HepB-CpG was compared with protection among 3,214 subjects receiving 3 doses of Engerix-B. Seroprotective anti-HBs levels were achieved in 90.0%–100.0% of subjects receiving HepB-CpG, compared with 70.5%–90.2% of subjects receiving Engerix-B (*4*–*7*). The body of evidence for the benefits of protection against HBV infection was deemed to be GRADE evidence type 2 (i.e., evidence from randomized controlled trials with important limitations, or exceptionally strong evidence from observational studies). The evidence type was downgraded for indirectness because immunogenicity was used as a surrogate for protection.

Safety profiles among 9,871 subjects receiving 2 or 3 doses of HepB-CpG were compared with those among 4,385 subjects receiving 3 or 4 doses of Engerix-B. Among subjects receiving HepB-CpG, 45.6%, 5.4%, and 0.27% experienced a mild adverse event, serious adverse event, or cardiovascular event, respectively. Among subjects receiving Engerix-B, 45.7%, 6.3%, and 0.14% experienced a mild adverse event, serious adverse event, or cardiovascular event, respectively (*1*,*4*–*9*). The body of evidence assessing adverse events was deemed to be GRADE evidence type 1 (evidence from randomized controlled trials, or overwhelming evidence from observational studies).

## Rationale

Based on the available immunogenicity evidence, a 2-dose schedule (0, 1 month) of HepB-CpG will be efficacious for the prevention of HBV infection. The risk for adverse events, including cardiovascular adverse events, was reviewed and will be monitored. The benefits of protection with 2 doses administered over 1 month make this an important option for prevention of HBV.

## ACIP Recommendations

HepB-CpG may be used as a HepB vaccine in persons aged ≥18 years recommended for vaccination against HBV (Box) (*2*).

BOX **Adults who are recommended to receive hepatitis B vaccine**

- Persons at risk for infection through sexual exposure
  - Sex partners of hepatitis B surface antigen (HBsAg)–positive persons
  - Sexually active persons not in a long-term, mutually monogamous relationship
  - Persons seeking evaluation or treatment for a sexually transmitted infection
  - Men who have sex with men
- Persons with a history of current or recent injection drug use
- Persons at risk for infection by percutaneous or mucosal exposure to blood
  - Household contacts of HBsAg-positive persons
  - Residents and staff of facilities for developmentally disabled persons
  - Health care and public safety personnel with reasonably anticipated risk for exposure to blood or blood-contaminated body fluids
  - Hemodialysis patients and predialysis, peritoneal dialysis, and home dialysis patients
  - Persons with diabetes mellitus aged <60 years and persons with diabetes mellitus aged ≥60 years at the discretion of the treating clinician
- International travelers to countries with high or intermediate levels of endemic HBV infection (HBsAg prevalence ≥2%)
- Persons with hepatitis C virus infection, persons with chronic liver disease (including, but not limited to, those with cirrhosis, fatty liver disease, alcoholic liver disease, autoimmune hepatitis, and an alanine aminotransferase [ALT] or aspartate aminotransferase [AST] level greater than twice the upper limit of normal)
- Persons with human immunodeficiency virus infection
- Incarcerated persons
- Other persons seeking protection from hepatitis B virus infection (even without acknowledgment of a specific risk factor)

## CDC Guidance for Use

**Interchangeability and dosing schedule.** Data are limited on the safety and immunogenicity effects when HepB-CpG is interchanged with HepB vaccines from other manufacturers. When feasible, the same manufacturer’s vaccines should be used to complete the series (*10*). However, vaccination should not be deferred when the manufacturer of the previously administered vaccine is unknown or when the vaccine from the same manufacturer is unavailable (*10*).

The 2-dose HepB vaccine series only applies when both doses in the series consist of HepB-CpG. Series consisting of a combination of 1 dose of HepB-CpG and a vaccine from a different manufacturer should consist of 3 total vaccine doses and should adhere to the 3-dose schedule minimum intervals of 4 weeks between dose 1 and 2, 8 weeks between dose 2 and 3, and 16 weeks between dose 1 and 3. Doses administered at less than the minimum interval should be repeated. However, a series containing 2 doses of HepB-CpG administered at least 4 weeks apart is valid, even if the patient received a single earlier dose from another manufacturer.

**Special populations.** There are no clinical studies of HepB-CpG in pregnant women. Available human data on HepB-CpG administered to pregnant women are insufficient to inform assessment of vaccine-associated risks in pregnancy. Until safety data are available for HepB-CpG, providers should continue to vaccinate pregnant women needing HepB vaccination with a vaccine from a different manufacturer.

**Postvaccination serologic testing**. To assess response to vaccination and the need for revaccination, postvaccination serologic testing 1–2 months after the final dose of vaccine is recommended for certain persons following vaccination (e.g., hemodialysis patients, HIV-infected and other immunocompromised persons, health care personnel, and sex partners of HBsAg-positive persons) (*2*). Postvaccination serologic testing should be performed using a method that allows determination of the protective level of anti-HBs (≥10 mIU/mL) (*2*). Persons with anti-HBs <10 mIU/mL following receipt of 2 doses of HepB-CpG should be revaccinated. Revaccination may consist of administration of a second complete HepB vaccine series followed by anti-HBs testing 1–2 months after the final dose. Alternatively, revaccination may consist of administration of an additional single HepB vaccine dose followed by anti-HBs testing 1–2 months later (and, if anti-HBs remains <10 mIU/mL, completion of the second HepB vaccine series followed again by anti-HBs testing 1–2 months after the final dose) (*2*). Administration of more than two complete HepB vaccine series is generally not recommended, except for hemodialysis patients (*2*). HepB-CpG may be used for revaccination following an initial HepB vaccine series that consisted of doses of HepB-CpG or doses from a different manufacturer (*11*). HepB-CpG may also be used to revaccinate new health care personnel (including the challenge dose) initially vaccinated with a vaccine from a different manufacturer in the distant past who have anti-HBs <10 mIU/mL upon hire or matriculation (*2*).

**Precautions and contraindications**. Before administering HepB-CpG, health care providers should consult the package insert for precautions, warnings, and contraindications. Adverse events occurring after administration of any vaccine should be reported to the Vaccine Adverse Event Reporting System (VAERS). Reports can be submitted to VAERS online, by fax, or by mail. Additional information about VAERS is available by telephone (1-800-822-7967) or online (https://vaers.hhs.gov).

## Future Considerations

Postlicensure surveillance studies and additional data pertaining to the use of HepB-CpG will be reviewed by ACIP as they become available, and recommendations will be updated as needed. Future economic analyses might inform cost-effectiveness considerations of HepB-CpG, including its use among persons at an increased risk for vaccine nonresponse.

## Footnotes

* ACIP is chartered as a federal advisory committee that provides expert external advice and guidance to the Director of CDC on use of vaccines and related agents for the control of vaccine-preventable diseases in the U.S. civilian population. ACIP recommendations adopted by the CDC Director become agency guidelines on the date published in *MMWR*.

† The ACIP Hepatitis Vaccines Work Group comprises professionals from academic medicine (family medicine, internal medicine, pediatrics, obstetrics, infectious disease, occupational health, and preventive medicine specialists), federal and state public health entities, and medical societies.
